# Lost in translation: why pharmacological trials keep failing in acute spinal cord injury

**DOI:** 10.1055/s-0046-1827054

**Published:** 2026-08-13

**Authors:** Abelardo Q. C. Araujo

**Affiliations:** 1 Universidade Federal do Rio de Janeiro, Instituto de Neurologia Deolindo Couto, Rio de Janeiro RJ, Brazil.; 2 Universidade Federal do Rio de Janeiro, Faculdade de Medicina, Rio de Janeiro RJ, Brazil.; 3 Universidade Federal Fluminense, Programa de Pós-Graduação em Neurologia e Neurociências, Rio de Janeiro RJ, Brazil.

**Keywords:** Spinal Cord Injuries, Neuroprotection, Translational Medical Research, Treatment Outcome, Methylprednisolone, Riluzole

## Abstract

Despite four decades of translational research, no pharmacological agent has been approved for acute traumatic spinal cord injury (SCI) based on class-I evidence. The current narrative review critically examines the seven agents evaluated in phase-II to -III clinical trials—methylprednisolone (MPSS), GM-1 monosialotetrahexosylganglioside, minocycline, riluzole, anti-Nogo-A antibody NG101, VX-210, and granulocyte colony-stimulating factor (G-CSF)—with emphasis on quantitative effect estimates, confidence intervals, and mechanisms of translational failure. Polylaminin, under phase-I evaluation approved by the Brazilian Health Regulatory Agency (Agência Nacional de Vigilância Sanitária, ANVISA, in Portuguese), is discussed in the context of disproportionate public communication relative to the stage of evidence; available human data are limited to a small, uncontrolled, unreviewed pilot study insufficient for efficacy inferences. High-dose MPSS conferred no motor recovery benefit in a meta-analysis of 1,863 participants and was significantly associated with gastrointestinal hemorrhage (odds ratio [OR] = 2.07; 95%CI: 1.02–4.20) and respiratory infections (OR = 1.73; 95%CI: 1.12–2.68). Furthermore, GM-1 ganglioside, G-CSF, and VX-210 failed to meet the prespecified primary endpoints. Minocycline produced inconclusive results in an underpowered phase-II study. Riluzole (Riluzole in Spinal Cord Injury Study [RISCIS]), which was prematurely terminated at 55% of enrollment due to the coronavirus disease 2019 (COVID-19) pandemic, showed a significant motor benefit in patients classified as grade C according to the American Spinal Injury Association (ASIA) Impairment Scale (AIS) (+8.0 points; 95%CI: 1.5–14.4; 2-tailed *p* = 0.034). The Nogo Inhibition in Spinal Cord Injury (NISCI) trial (2025), the most methodologically rigorous SCI trial to date, demonstrated no primary endpoint benefit but identified functional signals in motor-incomplete injuries. In total, six structural domains of translational failure are identified and methodological solutions proposed.

## INTRODUCTION

Acute traumatic spinal cord injury (SCI) affects between 250 thousand and 500 thousand individuals annually worldwide, with an estimated incidence of 40 cases per million per year in Brazil. 1 The primary mechanical injury triggers a complex secondary cascade—encompassing glutamate-mediated excitotoxicity, lipid peroxidation, calcium influx, microvascular ischemia, microglial activation, and oligodendroglial apoptosis—that unfolds over hours to days following the initial insult, providing the theoretical rationale for pharmacological intervention. Yet, despite this compelling biological rationale and more than four decades of translational research, no pharmacological agent has achieved regulatory approval based on class-I evidence for acute SCI. This paradox—robust preclinical efficacy systematically failing to replicate in controlled human trials—represents one of the most consequential methodological challenges in contemporary clinical neuroscience, accompanied by repeated cycles of premature public enthusiasm, adoption of unvalidated treatments, patient harm, and misallocation of research resources. The current article critically examines the main pharmacological therapies that have reached phases II or III of the clinical evaluation in acute SCI, with emphasis on quantitative effect estimates, 95%CIs, available meta-analyses, and the structural determinants of translational failure. The phenomenon of disproportionate public communication of preliminary findings and its measurable consequences for patients is addressed, and biomarker-guided strategies are discussed as requirements for future progress.

## LITERATURE SEARCH

The present work is a narrative review. The scope is defined by a concrete criterion: all pharmacological agents that have reached phases II or III of the clinical evaluation in adult humans with acute traumatic SCI were included. This criterion is self-delimiting and exhaustive; the seven agents discussed constitute the complete set of compounds tested in controlled clinical trials for this indication. Relevant preclinical studies, systematic reviews, and meta-analyses are cited when pertinent to translational analysis. Case reports, nonpharmacological interventions, and single-arm studies without concurrent controls were excluded unless specifically discussed in the context of early-stage evidence.

A structured search was conducted in the PubMed, EMBASE, and the Cochrane Central Register of Controlled Trials (CENTRAL), covering publications from January 1980 to January 2026, using the terms *spinal cord injury* AND ( *pharmacological therapy* OR *clinical trial* OR *neuroprotection* OR *randomized controlled trial* ), combined with agent-specific terms for each drug reviewed.

## METHYLPREDNISOLONE (MPSS): THE NASCIS CONTROVERSY

The biological rationale for MPSS in acute SCI was established by Hall and Braughler, demonstrating that supraphysiological doses inhibit post-traumatic lipid peroxidation, preserve regional spinal cord blood flow, and attenuate calcium influx in feline contusion models. 2 Critically, treatment in these models was initiated immediately after injury—while the animal remained under anesthesia—a condition radically different from clinical reality.

The 1984 National Acute Spinal Cord Injury Study (NASCIS I) randomized 330 patients to two MPSS regimens without a placebo group, yielding no significant difference between arms. 3 The NASCIS II, in 1990, enrolled 487 patients comparing high-dose MPSS (30 mg/kg bolus + 5.4 mg/kg/h for 23 h) with naloxone or placebo. 4 The prespecified primary analysis—across all 487 patients—found no statistically significant difference for motor or sensory recovery. A post-hoc subgroup restricted to patients treated within 8 hours demonstrated improved motor scores (+4–5 points), but this window was not prespecified in the protocol and was not corrected for multiple comparisons. Finally, the NASCIS III, in 1997, compared 24 versus 48-hour MPSS in 499 patients; the prespecified primary analysis was again negative ( *p* = 0.09 at 6 weeks; *p* = 0.07 at 6 months). 5 A post-hoc subgroup restricted to patients treated between 3 and 8 hours showed a benefit for 48-hour MPSS (+7.2 points, *p* = 0.008), without multiple-comparisons correction.

The meta-analysis by Liu et al. 6 analyzed 16 studies comprising 1,863 participants using random-effects models, finding no association between high-dose MPSS and improved motor scores in RCTs ( *p* = 0.84) or observational studies ( *p* = 0.44), nor with the grade of recovery on the American Spinal Injury Association (ASIA) Impairment Scale (AIS) ( *p* = 0.53) or sensory recovery ( *p* = 0.07). 6 Conversely, MPSS was significantly associated with higher rates of gastrointestinal hemorrhage (OR = 2.07; 95%CI: 1.02–4.20; *p* = 0.04) and respiratory infection (OR = 1.73; 95%CI: 1.12–2.68; *p* = 0.01). The adoption of MPSS as the “standard of care”—driven by the widespread interpretation of post-hoc NASCIS II data as establishing the first treatment for SCI—resulted in decades of patients receiving a treatment that conferred no motor benefit and increased risk of infection and gastrointestinal complications. 7 Most contemporary neurosurgical guidelines no longer recommend routine MPSS for acute SCI.

## GM-1 GANGLIOSIDE: THE PITFALL OF SMALL PILOT STUDIES

The Sygen Multicenter Acute Spinal Cord Injury Study represents a textbook case of a promising pilot study failing to replicate in a large, adequately powered trial. Geisler et al. 8 randomized 37 patients to GM-1 or placebo; the primary endpoint was not significant ( *p* = 0.088), but a post-hoc subgroup (n = 28) showed significant improvement in functional recovery (50 vs. 7.1%, *p* = 0.034). 8 This limited exploratory signal was extrapolated to a Phase III trial enrolling 760 patients. 9 The pre-specified primary endpoint—percentage of “marked recovery” (≥ 2 Benzel grades at 26 weeks)—was negative. The high-dose arm was suspended by the data safety monitoring board at the midpoint. The Sygen trial remains the largest SCI pharmacological study ever conducted, and its unequivocal negative result serves as a cautionary lesson about premature scale-up of underpowered pilot data driven by post-hoc subgroup signals.

## MINOCYCLINE: INCONCLUSIVE PHASE-II EVIDENCE

Minocycline inhibits microglial activation, reduces proinflammatory cytokines, blocks matrix metalloproteinases, and suppresses apoptotic pathways. Multiple murine contusion studies demonstrated histological and functional improvement, but independent replication attempts using standardized National Institutes of Health (NIH) protocols failed to reproduce the original positive results.

The only published RCT randomized 52 patients (27 to minocycline, 25 to placebo) and achieved plasma concentrations consistent with those observed in efficacious animal studies. 10 The primary endpoint—total motor score (TMS) recovery at 1 year—did not reach significance (difference: +6 points; 95%CI: −3–14; *p* = 0.20). Cervical injuries showed a nonsignificant trend (+14 points; 95%CI: 0–28; *p* = 0.05; n = 25). This trial was designed as an exploratory study for phase-III sample-size estimation; no adequately-powered multicenter phase-III trial of minocycline in acute SCI has been completed.

## RILUZOLE: INTERRUPTED EVIDENCE AND RESIDUAL SIGNAL

Riluzole, an United States Food and Drug Administration (FDA)-approved benzothiazole for amyotrophic lateral sclerosis, exerts dual neuroprotective actions by blocking voltage-gated sodium channels and inhibiting presynaptic glutamate release. Wu et al. demonstrated that riluzole administered up to 3 hours after cervical contusion in rats provided neuroprotection, improving locomotor distance, preserving myelin, and reducing lesion area. 11 Pharmacokinetic substudies in SCI patients demonstrated that acute SCI substantially alters riluzole disposition relative to amyotrophic lateral sclerosis (ALS) patients, implying that ALS-validated dosing regimens may be pharmacologically suboptimal in SCI. 12

The Riluzole in Spinal Cord Injury Study (RISCIS; NCT01597518) was an international, multicenter, double-blind, placebo-controlled, adaptive Phase III study designed to randomize 351 patients with traumatic cervical SCI (C4–C8, AIS A–C) within 12 hours of injury to riluzole or placebo. 13 A preplanned interim analysis had recommended continuation of the study. The trial was terminated by the COVID-19 pandemic in 2021, achieving 55% of planned enrollment (n = 193; n = 137 had complete 180-day outcome data).

The primary analysis—change in upper extremity motor score (UEMS) at 180 days—did not reach the prespecified efficacy threshold (+1.76 points; 95%CI: −2.54–6.06), an expected result given severe underpowering. In prespecified secondary subgroup analyses, riluzole was associated with a significant gain in TMS in AIS C patients (+8.0 points; 95%CI: 1.5–14.4; 2-tailed *p* = 0.034; originally 1-tailed *p* = 0.017, which was prespecified in the protocol).

A reanalysis using the Global Statistical Test (GST) integrating the ASIA total motor score, Spinal Cord Independence Measure (SCIM), and 36-item Short Form Survey (SF-36) physical component scale demonstrated superior median rank sum for riluzole versus placebo ( *p* = 0.04), corresponding to a 58% probability of superior overall outcome, with the largest benefit in AIS A patients (GTE = 0.16; 95% CI: 0.01–0.31; *p* = 0.02). 14 A meta-analysis encompassing five riluzole studies showed a consistent nonsignificant trend toward motor benefit. 15 The evidence base justifies an adequately powered dedicated Phase III trial in AIS C patients.

## ANTI-NOGO-A ANTIBODY NG101 (NISCI TRIAL)

Nogo-A, a transmembrane protein encoded by RTN4 and highly expressed by oligodendrocytes and central nervous system (CNS) neurons, is a potent inhibitor of axonal growth and regeneration. Neutralization by specific antibodies in murine models produced corticospinal tract sprouting, de novo synapse formation, and functional improvement across multiple independent laboratories. Among SCI pharmacological agents, anti-Nogo-A has published peer-reviewed primate-model validation of the specific compound subsequently tested in a clinical trial. Freund et al. demonstrated efficacy in cervical hemisection in rhesus macaques. 16

The Nogo Inhibition in Spinal Cord Injury (NISCI) trial was a phase-IIb, double-blinded, placebo-controlled study conducted at 13 European specialized SCI centers, published in 2025 (NCT03935321). 17 The trial enrolled 129 patients with acute traumatic cervical SCI (C1–C8, AIS A–D), randomized 4 to 28 days postinjury to receive six intrathecal injections of NG101 (45 mg) or placebo over 24 weeks, using an unbiased recursive partitioning (URP) stratification approach. Three patients were excluded postrandomization (two withdrew consent, one due to protocol violation), yielding a full analysis set of 126 patients (78 NG101; 48 placebo).

The primary endpoint—UEMS change at 168 days—was not significant (+1.37 points; 95%CI: −1.44–4.18). Safety profiles were comparable (serious adverse events: 14 vs. 13%; no treatment-related fatalities). Post-hoc subgroup analyses identified significant improvement in the SCIM self-care subscore in motor-incomplete cervical injuries ( *p* < 0.05), and cerebrospinal fluid (CSF) neurofilament light chain (NfL) and magnetic resonance imaging (MRI) tissue bridges correlated with neurological outcomes. These findings support the phase-Ib development of NG004, a second-generation anti-Nogo-A antibody.

## VX-210 (CETHRIN/BA-210): RHO PATHWAY INHIBITION AND FUTILITY

The VX-210, also known as *cethrin* or *BA-210* , is a recombinant fusion protein designed to inactivate Rho-A GTPase through epidural application, targeting intracellular mediators of growth cone collapse. A Phase I/IIa dose-escalation study in approximately 48 patients suggested signals of neurological improvement in cervical SCI. 18 The subsequent pivotal phase-IIb/-III trial failed to meet its primary efficacy endpoint and was halted for futility. A notable methodological concern is that the pivotal trial data were neither independently peer-reviewed nor published, precluding full evaluation of design quality and adverse event profiles—an absence of transparency that limits independent scientific assessment.

## GRANULOCYTE COLONY-STIMULATING FACTOR (G-CSF): PHASE-III FAILURE

The G-CSF demonstrated neuroprotective effects in animal SCI models by inhibiting oligodendroglial apoptosis, modulating inflammation, and activating the JAK2/STAT3 anti-apoptotic pathway. Phase I/IIa studies confirmed safety and feasibility in 16 patients. 19 A nonrandomized prospective study of 41 patients showed neurological improvement compared with historical controls, but without randomization causal inference is precluded. 20

The only Phase III RCT, in 2021, 21 randomized 88 patients with cervical SCI (AIS B or C) within 48 hours to G-CSF (400 µg/m ^2^ /day for 5 days IV) or placebo. The primary endpoint—TMS change at 3 months—was not significant (+2.3 points; 95%CI: −2.1–6.8; *p* = 0.31). 21 Exploratory subgroup analyses identifying trends in C4 to C5 injuries and in elderly patients ( *p* = 0.07 each) failed to meet multiple-comparisons adjustment. A key methodological concern is the risk of assessor unblinding due to treatment-induced leukocytosis.

## POLYLAMININ: BIOLOGICAL PLAUSIBILITY AND THE LIMITS OF THE AVAILABLE EVIDENCE

Polylaminin (polyLM) is a polymeric form of laminin developed by the Coelho-Sampaio group at Universidade Federal do Rio de Janeiro (UFRJ) designed to mimic native basement membrane organization and serve as a permissive substrate for axonal growth. 22 The seminal 2010 preclinical study demonstrated significant locomotor improvement following intraparenchymal polyLM injection after complete thoracic transection in rats, with retrograde axonal tracing documenting fibers crossing the transection site; nonpolymerized laminin produced no comparable benefit, demonstrating the specificity of the polymeric form. A 2025 longitudinal study in six dogs with chronic thoracic SCI reported statistically significant improvements in gait scores, although the simultaneous administration of glial cell line-derived neurotrophic factor (GDNF) and chondroitinase ABC precludes isolation of polyLM's contribution, and the absence of concurrent controls limits causal inference. 23

The only available human data derive from an open-label, single-arm pilot study in eight AIS A patients, available exclusively as an unreviewed preprint on medRxiv since February 2024. 24 From that total, two patients (25%) died in the early postintervention period from causes that the preprint authors attributed to their underlying condition. Of 6 evaluable survivors, AIS grade conversion from A to C or D was reported in 5 (83%) at 1 year, with motor score improvement in all survivors.

These results require cautious interpretation in light of several methodological limitations: reported rates of spontaneous motor recovery in AIS A injuries ranged from 5 to 20% over 1 year in published cohorts, although direct comparison is limited by differences in patient selection and baseline assessment methods; baseline neuroimaging data sufficient to stratify individual recovery probability were not systematically reported in the preprint; n = 6 is statistically insufficient for any inference about efficacy; and the absence of randomization, blinding, and concurrent controls makes it impossible to disentangle treatment effect from spontaneous recovery, surgical decompression effects, and confounders.

The 2026 Brazilian Health Regulatory Agency (Agência Nacional de Vigilância Sanitária, ANVISA, in Portuguese) phase-I approval authorizes a safety study in a small number of acute SCI patients and does not constitute evidence of clinical efficacy. PolyLM's biological rationale is genuine, and its rodent preclinical data are promising, but the trajectory from compelling animal results to clinical efficacy has failed systematically for every agent reviewed in this article. A rigorous phase-I–II sequence is the only scientifically and regulatorily valid path forward.

## SCIENTIFIC HYPE IN SCI RESEARCH: MECHANISMS, CONSEQUENCES, AND RESPONSIBILITY

The disproportionate and premature amplification of preliminary findings before adequate validation—also termed scientific hype—is particularly prevalent in neurological conditions for which effective treatments are lacking. The hype cycle in regenerative medicine for SCI follows a recurrent pattern: animal results are reported with language that suppresses methodological caveats; the media amplify the narrative; patients exert pressure for access before validation; and subsequent well-designed trials produce neutral or negative results, generating frustration and mistrust in science.

The MPSS represents the field's most extensively documented example. Post-hoc subgroup data from NASCIS II were widely interpreted in 1990 as establishing “the first treatment for SCI,” generating intense media coverage that transformed an exploratory, uncorrected secondary analysis into a global standard of care adopted for more than two decades. During that period, thousands of patients received a treatment that, as the best subsequent evidence indicates, conferred no net motor benefit and increased infectious and gastrointestinal complications—a measurable clinical cost with real adverse events in real patients.

The most recent and socially prominent episode involves polyLM in Brazil. Videos released in 2025, showing SCI patients performing voluntary movements, generated widespread online dissemination with tens of millions of views, describing a “Brazilian cure for paralysis” without adequate scientific context regarding the experimental stage, minimal patient numbers, absence of controls, or the fact that available data exist exclusively as an unreviewed preprint. Observable consequences followed: patient associations reported large volumes of inquiries; clinicians were approached for compassionate-use injections outside approved protocols; and individuals with chronic SCI travelled to Brazil seeking treatment, in some cases in unregulated settings.

The January 2026 ANVISA phase-I approval was widely mischaracterized in media coverage as proof of efficacy. The absence of peer-reviewed human data at the time of widespread public dissemination—with public communication preceding independent editorial scrutiny by years—illustrates a pattern in which the sequence of scientific validation is inverted, and the responsibility for ensuring proportionate communication of preliminary findings rests with researchers and institutions alike.

## MECHANISMS OF TRANSLATIONAL FAILURE: A STRUCTURED ANALYSIS

The systematic failure of pharmacological agents in SCI reflects structural and recurrent problems across six domains, summarized in Table 1 . 1 2 3 4 5 11 12 14 17 25 The first and most fundamental is the therapeutic window discrepancy: treatment in animal studies is administered within minutes of injury while the animal remains under anesthesia, whereas clinical trials enroll patients at a median of 3 to 12 hours postinjury. For mechanisms critically operative within the first hours—lipid peroxidation, excitotoxicity, calcium influx—the therapeutic window may be irreversibly closed by the time clinical treatment begins.

**Table 1:** Structural domains of translational failure in acute spinal cord injury pharmacology and proposed methodological solutions

Domain	Core mechanism of failure	Proposed methodological solution
1. Therapeutic window discrepancy	Animal treatment invariably initiated within minutes of injury (animal under anesthesia), whereas clinical trials enroll patients at a median of 3–12 h postinjury, with some protocols accepting up to 48 h. 2 3 4 5 11	Develop preclinical models incorporating treatment delays that mirror clinical reality; establish and validate clinically achievable therapeutic windows; prioritize agents with demonstrated efficacy beyond the 3-hour window.
2. Interspecies biological differences	Corticospinal tract runs through the dorsal funiculus in rodents versus the lateral funiculi in humans. White-to-gray matter ratio, CPG capacity, and spinal plasticity mechanisms differ substantially across species.	Require NHP validation before Phase III initiation; use STAIR-adapted multi-species models; complement rodent data with translational NHP studies.
3. Lesion heterogeneity	Human SCI is heterogeneous in mechanism, completeness (AIS A–D), neurological level, and comorbidities. Preclinical models employ standardized injuries in young, healthy male rodents. 1 25	Adopt biomarker-guided patient stratification (CSF NfL, MRI tissue bridges); implement population-enrichment designs targeting biologically coherent subgroups.
4. Preclinical methodological limitations	Publication rates exceeding 80–90% positive results are biologically implausible. Absent assessor blinding, inadequate randomization, insufficient sample sizes, and failure to require independent replication. 25	Implement STAIR-equivalent guidelines for SCI; mandate independent laboratory replication before clinical escalation; require publication of negative results.
5. Inadequate clinical endpoints	Total ASIA motor score treats a 0→1 gain equivalently to a 4→5 gain, creating systematic insensitivity for functionally meaningful change. MCID not pre-defined in most trials. 14 17	Pre-specify MCID for all endpoints; adopt multidimensional composite endpoints (GST: ASIA motor + SCIM + SF-36 Physical); incorporate SCIM as co-primary outcome.
6. Dynamic acute-phase pharmacokinetics	Neurogenic shock, tissue ischemia, and plasma protein redistribution dynamically modify drug clearance and bioavailability. Rarely adequately characterized before phase III. 12	Mandate pharmacokinetic sub-studies in all Phase II trials; use registry-based prospective PK data collection; adapt dosing regimens based on acute SCI-specific PK profiles.

Abbreviations: AIS, American Spinal Injury AssociationASIA Impairment Scale; ASIA, American Spinal Injury Association; CPG, central pattern generator; CSF NfL, cerebrospinal fluid neurofilament light chain; GST, global statistical test; MCID, minimal clinically-important difference; MRI, magnetic resonance imaging; NHP, nonhuman primate; PK, pharmacokinetics; SCIM, Spinal Cord Independence Measure; SCI, spinal cord injury; SF-36, 36-Item Short Form Survey; STAIR, Stroke Treatment Academic Industry Roundtable. Note: Superscript numbers refer to primary sources listed in the reference section.

The second domain concerns interspecies biological differences. The dominant lateral corticospinal tract in humans runs in the dorsolateral funiculus, in contrast to its dorsal column position in rodents—a fundamental anatomical difference with direct implications for injury mechanisms and regenerative capacity. The white-to-gray matter ratio and capacity of the central pattern generator differ substantially across species.

The third domain is the profound heterogeneity of human SCI in mechanism (contusion, fracture-dislocation, penetrating), completeness (AIS A–D), neurological level (∼ 50% cervical, ∼ 35% thoracic, ∼ 15% lumbosacral), and comorbidities, whereas preclinical models employ standardized injuries in young healthy male rodents.

The fourth domain—preclinical methodological limitations—is well documented: positive result rates exceeding 80 to 90% are biologically implausible given the condition's complexity. 25 Absent assessor blinding, inadequate randomization, insufficient sample sizes, retrospective outcome selection, and failure to require independent replication are systemic deficiencies. Effect sizes diminish with methodological quality—a dose-response relationship diagnostic of bias.

The fifth domain concerns inadequate clinical endpoints: the total ASIA motor score treats a 0-to-1 gain as equivalent to a 4-to-5 gain, creating systematic insensitivity to functionally meaningful change in incomplete injuries, and the minimal clinically-important difference (MCID) was not predefined in most trials.

The sixth domain is the dynamic pharmacology of the acute injury state, in which neurogenic shock, tissue ischemia, and plasma protein redistribution dynamically modify drug clearance and bioavailability—phenomena rarely adequately characterized before Phase III initiation. 12

## COMPARATIVE SUMMARY OF PHASE-II and -III TRIALS

Table 2 summarizes seven agents across eight trial entries evaluated in phase-II and -III clinical trials for acute traumatic SCI, arranged chronologically. The MPSS is represented by NASCIS II and III. Spanning four decades from NASCIS II (1990) to NISCI (2025), the table illustrates a consistent pattern of failure across mechanistically diverse compounds.

**Table 2:** Comparative summary of phase-II and -III clinical trials in acute traumatic spinal cord injury, arranged chronologically by primary publication date

Agent	Trial (year)	N	Design/Notes	Effect estimate (95%CI)	Conclusion	Funding
MPSS	NASCIS II (1990)	487	Phase-III RCT	Prespecified primary: negative. Post-hoc <8 h: +4–5 pts (uncorrected)	Negative	NIH/NINDS; drug: Upjohn
MPSS	NASCIS III (1997)	499	Phase-III RCT	Primary negative ( *p* = 0.09 at 6 weeks; *p* = 0.07 at 6 months). Post-hoc 3–8 h: +7.2 pts ( *p* = 0.008; not prespecified)	Negative	NIH/NINDS
GM-1 ganglioside	Sygen (2001)	760	Phase-III RCT	Primary endpoint (≥ 2 Benzel grades at 26 weeks): negative. High-dose arm suspended by DSMB	Negative	Fidia Pharmaceuticals (industry)
Minocycline	Casha et al. (2012)	52	Phase-II RCT; designed for phase-III sample-size estimation	TMS at 1 year: +6 points (95%CI: −3–14; *p* = 0.20). Cervical: +14 points (95%CI: 0–28; *p* = 0.05)	Inconclusive (underpowered)	CIHR; Alberta Paraplegic Foundation
VX-210 (Cethrin/BA-210) ‡	Phase IIb/III (terminated)	∼48 (I/IIa); pivotal N NR	Phase-I/-IIa dose-escalation; pivotal trial halted for futility; data not peer-reviewed	UEMS improvement: failed. Effect size not reported (trial halted for futility)	Negative (development discontinued)	BioAxone/Alseres (industry)
G-CSF	Koda et al. (2021)	88	Phase-III RCT	TMS at 3 months: +2.3 points (95%CI: −2.1–6.8; *p* = 0.31). C4–5 and elderly subgroups: *p* = 0.07 each	Negative	Chugai/JSPS KAKENHI (mixed)
Riluzole *	RISCIS (2023)	193 *	Phase-III adaptive RCT (55% enrolled; terminated COVID-19; interim analysis had recommended continuation)	UEMS at 180 days: +1.76 points (95%CI: −2.54–6.06). Prespecified AIS C TMS: +8.0 points (95%CI: 1.5–14.4; 2-tailed *p* = 0.034). GST reanalysis (2025): † *p* = 0.04	Inconclusive; signal in AIS C and motor-incomplete injuries	AO Foundation/DoD/Praxis (mixed public–private)
Anti-Nogo-A (NG101)	NISCI (2025)	126	Phase-IIb RCT; 13 European centers; URP stratification	UEMS at 168 days: +1.37 points (95%CI: −1.44–4.18; NS). SCIM self-care: post-hoc significant in motor-incomplete cervical injuries ( *p* < 0.05)	Negative primary; SCIM signal in motor-incomplete injuries	EU Horizon 2020/Swiss nonprofit academic consortium

Abbreviations: AIS, American Spinal Injury Association Impairment Scale; ASIA, American Spinal Injury Association; CIHR, Canadian Institutes of Health Research; COVID-19, coronavirus disease 2019; DSMB, data safety monitoring board; EU, European Union; G-CSF, granulocyte colony-stimulating factor; GST, global statistical test; JSPS, Japan Society for the Promotion of Science; MPSS, methylprednisolone sodium succinate; NASCIS, National Acute Spinal Cord Injury Study; NIH, National Institutes of Health; NISCI, Nogo Inhibition in Spinal Cord Injury; NINDS, National Institute of Neurological Disorders and Stroke; NR, not reported; RISCIS, Riluzole in Acute Spinal Cord Injury Study; RCT, randomized controlled trial; SCIM, Spinal Cord Independence Measure; TMS, total motor score; UEMS, upper extremity motor score. Notes: *Riluzole (RISCIS): terminated by COVID-19 pandemic at 55% enrollment (n = 193 of 351 planned); n = 137 patients had complete 180-day data; a preplanned interim analysis had recommended continuation. ^‡^ VX-210 (Cethrin/BA-210): Phase I/IIa dose-escalation study; 18 pivotal phase-IIb/-III trial halted for futility; data not independently peer-reviewed or published. ^†^ GST reanalysis: post-hoc analysis published by Fehlings et al.; 14 not the prespecified primary outcome of the RISCIS trial.

The two most recently completed trials, RISCIS (riluzole) and NISCI (NG101), reflect substantial advances in methodological rigor, incorporating adaptive designs, validated stratification methods, and multidimensional secondary outcomes. Both produced inconclusive primary results but identified biologically plausible signals in predefined subgroups of motor-incomplete injuries, providing the most credible basis to date for future population-enriched trial designs.

## FUTURE DIRECTIONS: TOWARD SMARTER TRIALS

The most promising near-term advance is the prospective use of validated biomarkers to enrich trial populations with patients most likely to respond. Cerebrospinal fluid and plasma NfL, quantified in the hyperacute phase, correlate strongly with injury severity and neurological outcome. The MRI tissue bridge quantification—measuring residual spinal cord tissue crossing the injury epicenter—stratifies patients by regenerative potential not captured by clinical examination alone, as demonstrated by the NISCI biomarker analyses. 17 These strategies have been prospectively validated and provide a practical template for future trials.

Practical implementation of biomarker-guided stratification in multicenter trials requires standardized infrastructure: centralized MRI reading with validated quantification protocols for tissue bridge measurement, uniform CSF collection within a defined time window postinjury with pre-analytical processing standards, and prospective biobanking for exploratory analyses. The NISCI trial demonstrated the feasibility of such an approach across 13 European specialized centers, providing an operational model that future trials can adopt and refine. Prespecified, biomarker-defined subgroups should be incorporated into adaptive enrichment designs, allowing sample-size reallocation toward populations that show biological signals of response.

The multifactorial nature of secondary SCI is unlikely to be addressed by any single pharmacological agent. The most intellectually coherent near-term framework integrates early neuroprotection (riluzole, systemic hypothermia) with regenerative strategies targeting inhibitory mechanisms (anti-Nogo-A, anti-repulsive guidance molecule A) and neuromodulation-based rehabilitation (epidural spinal cord stimulation), being evaluated using composite, multidimensional endpoints such as the GST. 26 Adaptive platform designs allowing prespecified modifications to sample size and subgroup focus based on accumulating data, and master protocol designs evaluating multiple agents within shared infrastructure, would dramatically reduce per-agent trial costs. An international SCI clinical trial network with standardized protocols and shared biorepositories represents a structural investment the field urgently requires.

Although this review focuses on pharmacological interventions, important parallel advances in nonpharmacological strategies deserve acknowledgment. Epidural electrical stimulation has demonstrated the capacity to enable voluntary movements in individuals with chronic motor-complete SCI, challenging long-held assumptions about the finality of complete injuries. 27 Brain–computer interfaces and activity-based rehabilitation programs represent additional modalities with emerging evidence of functional benefit. Future progress in SCI therapeutics will likely require integration of pharmacological neuroprotection, regenerative biologics, and neuromodulation within combinatorial trial designs—an approach that demands the same methodological rigor advocated throughout this review.

No advance in clinical trial design compensates for a deficient preclinical evidence base. Guidelines equivalent to those of the Stroke Therapy Academic Industry Roundtable (STAIR) adapted for SCI—mandating independent replication across at least two laboratories, multi-species models including non-human primates where mechanistically justified, inclusion of female and aged animals with comorbidities, mandatory publication of negative results, and pre-registration of study protocols—are nonnegotiable prerequisites. Prespecification of the MCID for all primary and secondary endpoints, and registry-based prospective pharmacokinetic data collection in the acute injury phase, are additional imperatives. Any promising candidate, regardless of biological plausibility, must follow the established phase-I–III sequence before clinical adoption can be justified.

In conclusion, after four decades of investigation, no pharmacological therapy has demonstrated Class I evidence of efficacy for acute traumatic spinal cord injury. High-dose methylprednisolone failed to show benefit in prespecified analyses, is associated with increased adverse events, and its historical adoption based on post-hoc data exposed thousands of patients to harm over more than two decades—the field's most extensively documented instance of premature clinical adoption.

Furthermore, GM-1 ganglioside, VX-210, and granulocyte colony-stimulating factor failed to meet prespecified primary endpoints in controlled trials. Minocycline remains inconclusive, based on a single underpowered Phase II study. Polylaminin has genuine preclinical promise, but available human data were limited to a small, uncontrolled, unreviewed pilot study that cannot support efficacy claims and must undergo rigorous randomized trials before any clinical adoption is justified. The most recent trials—RISCIS (riluzole) and NISCI (NG101)—reflect improved methodological rigor and produced biologically plausible signals in motor-incomplete injury subgroups, suggesting that progress depends on biomarker-guided patient enrichment, multidimensional outcome assessment, and adequately powered trials in biologically coherent populations.

The consistent pattern of failure across mechanistically diverse agents reveals six structural barriers: therapeutic window discrepancy, interspecies biological differences, lesion heterogeneity, preclinical methodological deficiencies, insensitive clinical endpoints, and inadequate acute-phase pharmacokinetic characterization. The history of SCI pharmacology does not demonstrate that neuroprotection is unattainable. It demonstrates that methodological rigor must precede therapeutic enthusiasm, and that disciplined communication of scientific uncertainty is an ethical obligation to patients who continue to wait.
